# Reports on the Diseases of London, and the State of the Weather from 1804 to 1816; Including Practical Remarks on the Causes and Treatment of the Former, and Preceded by a Historical View of the State of Health and Disease in the Metropolis in past Times, &c. &c. &c.

**Published:** 1820-01-01

**Authors:** 


### Medico-Chirurgical Journal;

OR,

### LONDON MEDICAL AND SURGICAL

### REVIEW.

Nec tibi quid liceat sed quid fecisse decebit
Occurrat mentemque domat respectus honesti. CLAUD.

No. 7.] JANUARY 1, 1820. [VOL. II.

### I.

*Reports on the Diseases of London, and the State of the Weather from* 1804 *to* 1816; *including Practical Remarks on the Causes and Treatment of the former, and preceded by a Historical View of the State of Health and Disease in the Metropolis in past Times, &c. &c. &c.* By THOMAS BATEMAN, M. D. F. L. S. &c. One volume Octavo, pp. 293. London, 1819.

A SURVEY of this boundless metropolis, and of the countless myriads of human beings which it every morning vomits forth from its dormitories, is well calculated to excite the most varied and vivid trains of reflection in the mind of the medical as well as the moral philosopher. A flourishing population may be compared to the human frame itself, in its progress to maturity. The whole system expands and strengthens, though all its parts are daily decaying and flying off as effete materials, no longer necessary for the growing fabric. It is so with the community at large; it appears, and really is, in perpetual bloom and vigour of youth, while the individuals of which it is composed, are hourly mingling, in thousands, with their mother earth!

Nature seems every where so anxious to perpetuate the human species, and indeed the whole animal and vegetable creation, that, under ordinary circumstances, the business of reproduction far exceeds the process of decay, until the maximum point is gained, when the vast machine checks its own velocity, by the developement of principles inherent in itself. Such principles have long been in full operation on certain spots of the earth's surface—for instance, in China, where the population is at a stand, or nearly so, and where Life and Death reign with equal sway. And as Youth is the season of hope, and joy, and every generous passion, so the balance of human happiness and enjoyment is clearly on the side of an increasing population. No man who has contemplated the frame of society in China and America, can be insensible to the truth of this remark.

It behoves the medical philosopher, therefore, to watch and study the various moral and physical agents which operate *collectively* as well as individually on the health of society; for, by such conduct only, can he obtain comprehensive views of disease, or correct principles on which to found the means of prevention and treatment.

It is true that Man, by the exertion of his intellectual faculties, can often counteract, in a considerable degree, the morbific agents around him; yet the great mass of society are the very sport of the elements that encompass them. We darken in our hue as we approach the Equator; we become of stunted and shrivelled growth in the vicinity of the Pole. Under the temperate, but variable skies of Europe, and especially of England, we are at the mercy of every blast; and thousands of us are annually beaten down by atmospherical commotions, like the corn in our fields, to rot in premature decay!

Although every class of society has, unfortunately, its own peculiar predispositions to disease, in the present state of the world; yet, it must be remembered, that the congregated masses of human beings, attracted or impelled to the metropolitan vortex by ambition, avarice, want, and all the stronger impulses of our nature, experience there a degree of wear and tear, both mental and corporeal, far beyond any thing observable in rural or provincial life. The conflicts of competition, with all its attendant anxieties; the sedentary habits; the unperflated abodes, and the insalutary occupations of so many thousands of people, must operate inimically on the health, and curtail the existence of a large proportion of the metropolitan population.

And this leads to the work under review. We entirely agree with Dr. Bateman in the great advantage which must result to medical science, and to public hygiene, " from faithful histories of the air, and the most obvious mutations in respect to its qualities, together with correct accounts of the several diseases, &c."* But we beg leave to enter our protest, *in limine*, against certain informalities

* Fothergill.

accompanying Dr. Bateman's work — informalities which are injurious to the true interest both of author and reader.

The pressure of public poverty, resulting from the long and exhausting conflict in which we were engaged, is now felt through every ramification of private life. No class of society suffers more, in this respect, than that of medical practitioners. A great proportion of them are forced to abridge the necessaries of life; and as books come rather under the denomination of luxuries, their circulation, or at least their sale, is rapidly diminishing. Men who formerly took in the most respectable works of the day, are now compelled to join in the Book-club, or subscribe to the Library, and thus have only a superficial and hasty perusal of books which they would, under better circumstances, carefully study and digest. Such being the case, we deem it a breach of public justice, for any author to republish his works, with title pages and *advertisements* that convey no hint of their being republications; but which, on the contrary, hold out every hope and expectation of their being original productions. The present volume is republished verbatim from the "Reports of Diseases at the Public Dispensary," inserted quarterly in the Edinburgh Medical and Surgical Journal, [a work which is deservedly in the hands of almost every medical reader] preceded by a short historical survey of thirty pages, and interspersed with a few appended lines, at long intervals, at the ends of some Reports. It is very true, that towards the close of the *Preface*, the real state of the case comes out; but then our money is irrecoverably gone; for no bookseller will take the work off our hands!

Neither do we object to the book, as a useless publication for all those who did *not* purchase the Reports in the Journal; but, surely Dr. Bateman cannot, with any show of reason, expect us to buy the same work twice. Let the republication be honestly and candidly stated in the title page, and also in the public advertisements; and then the purchasers of duplicates can have but themselves to blame. The evil of which we here complain has, of late, been growing; but it is one which no precedent can sanction, nor argument excuse.

From the nature of the case, we shall, in the following analysis, draw frequently on our own store of original observations and reflections; and hope, on the whole, to render this article one of no mean interest and utility.

1. *Historical Survey.* A comparison of former with present times will show that, notwithstanding the vast and still increasing extension of the metropolis, the ratio of mortality has decreased, while some tribes of disease have, as it were, become extinct. In 1697, the total mortality was 20,970; whereas, 100 years afterwards, viz. in 1797, and when, of course, the population was vastly augmented, the number of deaths only amounted to 17,014. This difference cannot be accounted for by any change in the *climate,* but by some principle of amelioration in the condition of the people themselves, in their modes of life, the construction of their abodes, and in the state of the earth's surface on which they reside.

Among the extinguished tribes we may enumerate PLAGUE, whose last visitation was made in 1665. With the plague, petechial, intermittent, and remittent fevers have nearly disappeared; for the present epidemic contagious fever, which is often attended with petechiæ, is a mere trifle, compared with the malignant fevers of earlier dates.

DYSENTERY, in former periods, committed such great ravages, as to be denominated by Graunt "the plague in the guts," and generally carried off 2000 annually. Rickets and scurvy, now almost unknown, or at least rarely fatal, were once very prevailing and mortal diseases.

We need not waste time in combating the idea of the smoak from coal, being a cause of the former unhealthiness in London. It is sufficient to say that the effect has ceased, while the [supposed] cause has increased. The great and efficient sources of sickness, at all periods and seasons of the year, in cities, in camps, and in fleets, may be traced to atmospherical vicissitudes, and atmospherical impregnations. From the *former,* we probably suffer as much now as at any preceding era, if not more. Scrofula and pulmonary complaints, alone, carry off at least a fourth of the population; but as riches and luxury have increased, so as to enable a great portion of those afflicted with the above-mentioned diseases to migrate from the "smoaky city" to the "pure air of the country," there to rest in peace for ever, the balance of *comparative* healthiness of London, in recent times, may be somewhat more apparent than real.

It is on the score of atmospherical impregnations that London, and all large towns in England, have rapidly improved, during the last fifty years in particular. The diseases by which the metropolis, in common with provincial cities, was commonly infested during and previous to the 17th century, were those which scourged our camps and fleets, when the *former* remained stationary for any time, so that filth might accumulate, and afford pabulum for miasmata, under the action of summer and autumnal suns; or where the neighbourhood of a marsh or a jungle supplied the place of filth. In respect to our fleets, it has been abundantly proved, that accumulations of filth in the hold, and on the lower decks of a vessel, will form, in all respects, a floating marsh; from which, under certain conditions of atmospheric temperature, febrific miasmata will be plentifully evolved, and scatter death throughout the crew.

A large town is but an extensive camp, or an immense fleet moored close together, with this inconvenience, that it has not the capability of loco-motion. As it cannot move *from* the sources of disease, it is necessary, therefore, that these sources should be cut off entirely, or quickly removed when once generated. It is on these two principles that Civic Hygiene has been successfully grounded. Cloacæ, drains, and common sewers, have been constructed; and water has been made to permeate, by innumerable conduits, through every street and dwelling, like arteries through the body, by which all impurities are swept along subterranean exits to the great reservoir in the ocean. The streets, too, are flagged, paved, and kept clean, so that no dirt can accumulate above, nor exhalation escape from below. In exact proportion as ventilation and cleanliness were observed, the health of the inhabitants improved. The great fire in 1666, though a tremendous affliction at the time, was, like most other evils, productive of ultimate benefit. But, for a considerable period after the fire, the habit of throwing the refuse of victuals, &c. into the streets, which were otherwise badly paved and drained, together with the feeding of animals, such as hogs, goats, and poultry in them, as is proved by the royal ordinances forbidding such nuisances, contributed to keep up the unhealthy state of the metropolis.

"Projecting spouts, in narrow old streets, still poured their collected waters from the roofs of the houses, impetuously upon the dripping passengers; while in all the streets, large sign-boards hung across, by irons fixed to the fronts of the houses, which, in proportion to the abilities of the shop-keepers, were carried to extravagant degrees of ostentation, not only obstructing the view, but the free circulation of air, grating the ear with most discordant creaking as they swung to and fro in windy weather. The middle of the streets were paved with large pebbles, of all sizes and shapes, and worn by carriages into dangerous holes that retained the rains in too great quantities to suffer the streets to be called clean, except in extreme dry weather, when the dust was as troublesome as the dirt and wet."

Many of the narrower streets were altogether unpaved; the sewers were much neglected, and the drains all ran above ground. The internal economy of the habitations was on a par with the streets.

"The floors, says Erasmus, in a letter to Cardinal Wolsey's Physician, are commonly of clay, strewed with rushes, which are occasionally renewed; but underneath lies unmolested an ancient collection of beer, grease, fragments of fish, spittle, the excrements of dogs and cats, and every thing that is nasty."

Hentzner states, that even the floor of the presence-chamber of Queen Elizabeth, in Greenwich palace, "was covered with hay, after the English fashion."

Such being the state of things among the rich, we may imagine the filth and misery of the poor! There were, in fact, abundance of causes quite adequate to the generation of fevers of all kinds, and admirably adapted to propagate the same by contagion afterwards.

We have hinted before, that the decreasing ratio of mortality in London, corresponding with the improved state of the inhabitants, in respect to cleanliness, ventilation and comfort, was probably more apparent than real; a probability which Dr. Bateman does not seem to take into consideration. He acknowledges, it is true, that "a few diseases may be observed to have increased in number and fatality;" but does not appear aware that this circumstance can tend to the introduction of deception in the bills of mortality.

"But these [the increased number and fatality of certain diseases] appear to be less dependent on the physical than on the moral and political changes, which the country has undergone; and are to be attributed chiefly to the increase of manufactures, and consequently of the number of sedentary and otherwise unwholesome occupations; *to the augmentation of national wealth, and with it, of luxury and high feeding;* and to the *fluctuations in the conditions of life,* attendant on the spirit of *commercial* speculation. To the first of these sources we may probably, in part, ascribe the regular increase of consumption during the last century; to the second, the more inconsiderable, but scarcely less regular increase of *apoplexy*, palsy, and *sudden deaths,* as well as of the gout; and to the last, the more frequent occurrence of insanity in its different forms: and the increase of intemperance and vice, in a large and populous city, doubtless contributes much to the augmentation of all these diseases." P. 21.

The facts are here correctly stated, but we conceive that

Dr. Bateman has failed to draw a proper inference from them. There can be no doubt that, notwithstanding the temporary difficulties in which we are now involved, the quantum of national wealth, and the condition of the middling and lower orders of the people, are infinitely superior to what they were in former times. This increase of wealth, as daily observation proves, enables a vast proportion of those afflicted with chronic and visceral complaints, especially *pulmonary* diseases, to migrate into the country, and there, in general, to terminate their days. This is so decidedly the case in the summer, and particularly among the wealthier classes, that London is then almost completely expurgated of patients, as Dr. Bateman must well know; and even the doctors themselves are forced, at such seasons, to evacuate the metropolis, and pitch their tents in all directions of coast and country, to meet the demands of *ex urbe* invalids. It is evident that the deaths among this numerous class must subtract materially from the comparative ratio of mortality in the metropolis. It is also to be recollected that a great proportion of those chronic diseases which have so prodigiously augmented, of late, only shorten the average range of life a few years, from their occurring most commonly at and after middle age; so that although there may be twice as much actual disease existing now as there was one hundred years ago, the ratio of *mortality* will not shew it near so much as when acute epidemics prevailed, that spared neither age nor sex. Neither does Dr. Bateman take at all into consideration the decrease of mortality in small pox; a circumstance of the utmost importance, since ten deaths in *infancy* will alter the ratio of mortality more than one hundred in the decline of life.

From these and many other considerations, which will be presently alluded to, we are clearly of opinion that the diseases of the metropolis and other great towns have multiplied in number within the last half century; and that, although certain tribes of fatal endemics have nearly disappeared, the decrease of mortality, on the whole, has been rather numerical than real; the diseases of refinement and civilization being a considerable set off against the public hygiene accompanying these states. And this leads us naturally to an examination of those infirmities which, "like shades pursuing wealth and fame," have followed close on the heels of luxury, of war, and of commerce. Dr. Bateman enumerates among the growing evils of the day, gout, insanity, sudden deaths, apoplexy and palsy; to which he ought to have added, diseases of the heart and nervous system. We shall glance at these in rotation, commencing with the following extract from a recent medical publication.

"But among the wonderful variety of means by which Nature counteracts the repletion resulting from too much and too rich food, stands GOUT. This, though a severe disease in itself, is yet an undoubted remedy or preventive of numerous other and more fatal ones. After a course of luxurious living, of longer or shorter duration, according to peculiarity of constitution, the human machine can no longer bear the rich tide of nutriment which daily flows through the interior organs, without danger of some of its channels giving way, and suddenly snapping the thread of life, as happens in apoplexy, the bursting of blood-vessels, &c. Nature, alarmed, now adopts a severe but a salutary measure. She generally gives notice of the approach of her operation, by first deranging the function of the *stomach,* for a few days, with occasional premonitory sensations in other parts of the body, as coldness of the feet, &c. Then the storm bursts. A paroxysm of pain and irritation is kindled up on some extreme part of the body, and the whole constitution is kept, during a time, in a feverish and restless condition, while a daily and critical discharge by the skin and kidnies reduces the system to a certain point compatible with health, when a calm ensues—the functions of the stomach and other organs resume their accustomed tone, and the luxurious advocate of civic society returns to the pleasures of the table with renovated vigour."

Now if the author of the above passage be correct, not only have the causes of gout been increasing during the last century, but the means of cure, pretty generally resorted to of late years in that disease, must have contributed to swell the list of apoplexy, sudden deaths, &c. When the pain and irritation of gout are not suffered to be moderately expended on some member or part, at a distance from the vital centre; when, on the contrary, a violent commotion is raised in the system by internal remedies; or when the inflammation is suddenly arrested or repelled by external cold, then, in all probability, will the irritation be transferred to some interior organ or tissue, and there manifest itself, at some future day, in the shape of a chronic disease, especially of the brain, heart, or digestive organs.

We cannot wonder that the venders of nostrums should still endeavour to deceive the public, even at the expence of public health; but it is lamentable to see some physicians pertinaciously adhere to the most dangerous and erroneous doctrines and practices, in this prevalent disease, with their eyes wilfully shut against the light of truth! The overwhelming mass of awful examples and fearful facts which are daily accumulating, however, will, ere long, put down this class of public aggressors.

We would fain hope that the fundamental sources of gout, as laid in the indulgences of the table, are rather on the decline than on the increase. Intemperance in drink is certainly on the wane among the upper and middling classes of society, partly from the pressure of the times, and partly, we imagine, from a more enlightened appreciation of the danger and impropriety of those disgraceful habits. Upon the whole, we anticipate a diminution of this evil in itself, and a relinquishment of those "short, easy, and effectual" modes of cure which, in too many instances, pave the way for long, painful, and incurable diseases afterwards.

*Insanity.* Dr. Bateman appears to think that this disease has not greatly increased during the last half century; the average number in the metropolis stands pretty steadily, according to Dr. Willan, at about 2600, confined in public and private asyla. It may be true that the open and unequivocal forms of insanity may not have considerably augmented; but that the lighter shades, concealed under the mild epithets of hypochondriasis, melancholy, and nervous diseases, have multiplied amazingly, we have not the smallest doubt.

"Those of the superior order, says Dr. Willan, whom their friends consign to these mansions of security, are not the victims of disappointed ambition, or of inordinate affections; not dissipated females sunk to ruin by their extravagance, nor men who have lost their all in the whirlpools of St. James's Street; but residents nearer the Royal Exchange; some of them shattered by unhealthy climates, some by overstraining the faculties, both of body and mind, in the acquisition of wealth; some ruined by deceiving, and perhaps self-deceiving projectors; others by the most daring commercial speculations; and a few whose understandings have been overset by mistaken views of religion."

It will not be denied that all these causes have prodigiously increased during the last fifty years; even the last one on the list, for scepticism and fanaticism go hand in hand. While one set of illuminati almost deny the existence of a Deity; there is another, and a much more numerous class, who not merely, like the untutored Indian, "See God in clouds, and hear him in the wind;" but conceive that the finger of Providence guides every the minutest transaction of life, and who, of course, from being in supposed constant communication with the Almighty, and from being, as they imagine, most intimately acquainted with all his mysterious ways, become entangled in all manner of fanatical absurdities, till, in too many instances, the seat of reason gives way, and they are plunged into mental darknesss !* But we shall not dilate on this subject, as it will be taken up more regularly, ere long, in another Number of our Journal.

*Sudden Deaths.* These are referable chiefly, if not solely, to affections of the head, and of the heart. Of the former we shall speak presently.

It is well known that Corvisart considers diseases of the heart as next, in order of frequency, to those of the lungs. He assigns two principal causes for this frequency ; one is, *the constant action* of the heart, from the cradle to the grave. This, by the bye, appears to us a very ridiculous cause ; since the divine architect has adapted the central organ of the circulation to its function of perpetual motion, equally as well as the brain to alternate waking and sleep, or the stomach to periodical digestion and repose. The second class of causes, which he assigns for organic and functional derangements of the heart, is certainly one of extensive and fatal operation ; namely, the "play of the passions." The greater number of these, as joy, anger, love, ambition, &c. derange the healthy rhythm of the heart's action by accelerating its motions, and this acceleration of movement is much more prone to hurt the structure of the organ than an opposite state of the circulation. A fertile source of cardiac disease is also to be sought in the *lungs.* Every one knows the intimate connection which subsists between respiration and circulation. Any obstruction to the transit of blood through the pulmonary vessels must throw additional labour on the right ventricle and auricle of the heart ; and it is on this account that, in most cases of obstinate asthma, and of other chronic diseases of the lungs, now so prevalent, we find dilatation or some organic change in the right cavities of the heart.

But here it may be asked, how it comes to pass that diseases of the heart have only begun to excite much attention within the last fifteen or twenty years ? Several substantial reasons, we think, may be assigned for this. 1st. Because morbid anatomy was never cultivated with general

* See Haslam's Observations on Madness and Melancholy.

zeal, among all ranks of the profession, till within the period alluded to. The consequence was, *first*, that a great number died of diseases of the heart, where no suspicion was entertained of the true nature of the malady. *Secondly*, the various consequences of derangements of the heart, especially dropsical effusions into the different cavities, were too often considered as the primary diseases and sole causes of the patient's death; the organ of the circulation being overlooked, or carelessly examined, of which we have seen many instances.

But moreover, there is strong reason to believe that diseases of the heart have greatly increased, both in frequency and force, during the last twenty-five years. There never was a period like that, for intense excitement of all the more turbulent as well as the more depressing passions. No class of society in Europe escaped the mental pertubations which raged from the commencement of the French revolution till the late general peace. These commotions in our moral nature produced the most deleterious influence on the functions and structure of the heart; and it is much to be feared that the present frame and state of society are little calculated to diminish the evil. It is therefore our duty and interest to watch the progress of this direful class of human afflictions, in order that we may be able to recognise them when we meet them, and alleviate where we cannot cure.

Before quitting this subject we may hint our belief, founded on observation, that misplaced gout and rheumatism have lately tended to swell the list of cardiac affections; partly, or principally perhaps, from the charlatanneries which have mixed in the treatment of these constitutional diseases.

The rapid increase of APOPLEXY, in these later years, has now attracted the attention of all medical, and even common observers; nor do we think that the circumstance is at all accounted for, on the supposition of an increased repletion in the way of diet. In the fourth Number of this Journal it was shewn that, even in the aged and infirm, active aneurisms or enlargements of the heart were infinitely more prevalent than passive diseases of that organ. Now every man of observation must have remarked the distress occasioned in the head by derangements of the heart, and especially by active enlargements, accompanied as they are by inordinate action of the diseased organ. In two instances which lately fell under our notice, there was every reason to believe that the apoplectic attack was determined by active enlargement of the heart; and it was with much satisfaction that we lately read a strong confirmation of opinions which we had for some time entertained on this subject, by M. Bricheteau, of Paris, of which we shall take some notice here.*

Baglivi appears to have been the first who has recorded a case illustrative of this connection, in opening the body of Malpighi, who died of apoplexy. The left ventricle of the heart was found actively enlarged, but Baglivi drew no conclusion from this coincidence or consequence. Gibellini [*de quibusdam cordis affectionibus*] details a similar case, and Lieutaud another. But M. Richerand seems to be the first who distinctly traced apoplexy to aneurism of the heart. "L'ouverture des cadavres des personnes mortes d'apoplexie, says he, m'a prouvé que *l'excès de force du ventricule gauche est une disposition plus prochaine à l'apoplexie,* qu'un cou court, &c." *Nosog. Chir. tome 3.*

Legallois, a few years ago, read before the *Ecole de Medecine* a very curious case of apoplexy resulting from the inordinate force and action of the left ventricle of the heart. The subject was a woman, who felt a sense of suffocation on the least motion; her sleep was disturbed; she could only lie on the right side, and was subject to nasal hæmorrhages. She was cut off in the 25th year of her age by an apoplectic seizure. On dissection, an effusion of blood on the brain was found, while the *left* ventricle of the heart was so enlarged, and its parietes of such preternatural strength, as to leave no doubt in Legallois's mind that the inordinate action of the heart was the cause of the apoplexy.

Richerand has still more recently stated an instance in point, in the case of the late illustrious Cabanis, who perished by a fourth attack of apoplexy in the spring of 1819. The *left* ventricle of the heart was enlarged to thrice its volume, and its walls increased to triple their usual strength.

But it is M. Bricheteau who has given the greatest development to this interesting investigation. During two years of careful observation at the Hotel Dieu, where the cases were open to the inspection of all, he has been able to collect a great body of evidence in proof of the connection between cardiac aneurism and apoplexy, thirteen

* De l'influence de la circulation sur les fonctions cerebrales, et de la connexion de l'hypertrophie du cœur avec quelques lesions du cerveau. *Journal Complimentaire des Sciences Medicales, Juill. 1819.*

cases of which are detailed in the Journal before mentioned. We shall condense and translate the fourth case as a specimen.

*Case.* A woman, 50 years of age, after having been turned out of several hospitals, was received into the Hotel Dieu, in May, 1816. She had not slept, with any kind of comfort, for several months; was obliged to keep in the erect posture in bed; was harrassed with a sense of suffocation, and pain in the epigastric region. No pulsation of the heart to be felt in any part of the chest; pulse regular, feeble, and slow; breathing quick and laborious; constant restlessness and complaining; lower extremities œdematous. Although many of the characteristic symptoms of cardiac disease were absent, it was yet suspected by the medical attendants; and two blisters were applied to the thighs, in the hope of relieving the epigastric pain, of which she so bitterly complained, administering, at the same time, antispasmodics, digitalis, &c. A few days after her entrance into the hospital, she was found one morning hemiplegic of the left side, with a total suspension of the excruciating pain with which she had been so long harrassed. She now fell into a state of indifference as to her situation; became daily enfeebled; the left arm swelled, and, strange to say, the pulsations of the heart *now* became evident, though extremely irregular. She died ten days after the hemiplegic attack. On dissection, the heart was found so enlarged as to almost fill the left side of the thorax, the parietes of the left ventricle having acquired a great thickness, and the carneæ columnæ enormous dimensions. There was effusion of blood within the cranium.

The various other cases which Bricheteau has detailed, tend to the same point; and from what we have now brought forward, we think we may safely conclude that the late increase of diseases of the HEART has contributed considerably to the increase of APOPLEXY, and perhaps of many other disorders denominated nervous.

But it must be recollected that the same moral causes which originally deranged the function and ultimately the structure of the heart, acted primarily through the medium of the sensorium; the organ not only of sensation and perception, but of reflection, and all those complex intellectual operations which are so multiplied and overwrought in high states of civilization, and particularly in times of political commotion and mercantile speculation. If the moderate and regular exercise of an organ tends to maintain freedom of function and integrity of structure, it is equally certain that undue and irregular exertion of the same will sooner or later derange both. The brain then, in modern times, is one of those organs which have been *over-excited,* and consequently predisposed, in an unusual degree, to disease; and this predisposition is called frequently into action by the derangement of other organs, and especially of the heart.

It is to be hoped that these etiological observations will not be considered as digressions. They grow out of the very nature of the subject, and they have been left untouched by Dr. Bateman, though it was his duty to dwell minutely upon them. It is of much less consequence to explain to us the causes which have contributed to our present state of salubrity, and which are now firmly established, than to investigate the causes of insalubrity which still exist, and which are allowed to be increasing; yet, strange to say, Dr. Bateman has not considered these as worthy of any examination.

At the conclusion of the historical survey Dr. B. alludes to the change which has taken place in the comparative health of the different seasons themselves. It is easy to conceive that, having removed from the surface of the soil all pabulum for the extrication of noxious exhalations by the summer and autumnal suns, *these* seasons should now, in consequence thereof, become the most healthy periods of the year; while the cold and vicissitudes of winter and spring continue to operate with baneful influence on our frames, and render *those* quarters of the year most pregnant with sickness and death. These facts are so clear and easily accounted for, that we need not dilate upon them farther.

2. We now come to the re-published Reports, the long tables and dry meteorological details of which, we shall entirely pass over, in order to concentrate our remarks on such practical incidents as are scattered among them. These incidents, it is true, will often serve but as texts on which we shall hang original observations or practical researches of our own; a line of conduct which we have reason to know will not be unacceptable to many of our constituents.

*Rep.* 2. *Nov. to Feb.* 1805.* In this report there is a passage respecting pulmonic complaints which deserves remark.

* See *Ed. Journal* for all the reports, according to the time specified.

"These chiefly assumed the character of peripneumonia, and were attended with symptoms of debility, rather than of acute inflammatory excitement. As the disease advanced the cough became troublesome, the respiration difficult, an obtuse wandering pain was felt in the chest, and the voice was much impaired; the tongue was brown and much furred; the pulse above 100 and feeble, *but the strength was not severely depressed as in typhus.* In one case, the patient died on the fourth day, with the *symptoms just enumerated*; and, on examining after death, both lobes of the lungs were found of a dark purple colour, especially of the upper and back part, and resembled the appearance of the *liver*, rather than of the healthy lungs; the bronchial cells being filled with effused fluid. In general, the disease yielded to blisters with diaphoretics. Blood-letting on the one hand, and a cordial treatment on the other, were contra-indicated by the two opposite sets of symptoms; but in these unfortunate combinations *experience* rather warrants an approximation to the *cordial treatment* of typhus, than to the debilitating remedies of pneumonia."

In the first place, with the appearances on dissection, above related, before him, we wonder how Dr. Bateman could think of the cordial treatment of typhus. But he tells us that the deduction was warranted by *experience;* yet, in a note on this passage, he now informs us that it "was the result of the *doctrines*," then taught in the schools. Which statement are we to credit? The latter no doubt. Dr. Bateman, in coming into the depletory treatment after such experience, and after a great many others had proved the pernicious effects of the cordial modes of cure, must be contented with the humble title of a follower, and not an original promulgator of these improvements in medical science. The passage, however, forms a good beacon for future practitioners; and shews us how much safer it is to be guided by *post mortem* appearances than by theories, or even by symptoms during life.

In the 4th Report [May—August 1805] Dr. Bateman reiterates the old story of redundant secretion of *bile* being the cause of cholera morbus, both in this country and in hot climates. But the heterodox doctrine, broached some years ago, that inordinate secretion of bile made no part of the etiology of cholera morbus, is now confirmed by the most ample testimonies.* In our eastern dominions the disease has spread epidemically for thousands of miles, and consequently the most extensive field for observation

* *See the Works of Drs. Armstrong, Ayre, and Johnson.*

has there been laid open to the pathological observer. Dr. Steuart Anderson* has lately presented the medical world with many interesting particulars of this dreadful scourge. It is said to have originated in Bengal, and, radiating thence in both southern and western directions, it has visited, indiscriminately, camps, cities, and villages, sparing only the hill-forts.

It travelled at about the rate of fifteen or twenty miles a-day, and that generally against the course of the monsoon. The symptoms of cholera need not be described. On dissection the large interior veins were found so congested as to contain nearly all the blood of the body. The liver and spleen were gorged. The veins of the stomach and mesentery appeared as if forcibly injected; "and the minute vessels on the intestines had their ramifications displayed by their coloured contents, which cast a general tinge on these viscera similar to what is observed in incipient inflammation."

The stomach was found to contain some "muddy water," while the intestines were distended with flatus, excepting the colon and rectum, which were generally contracted. *No bile was found in any part of the primæ viæ, between the mouth and the anus.* Great *atmospherical vicissitudes* preceded the breaking out of this epidemic.

The treatment, as most generally pursued, and as recommended by the Medical Boards of Calcutta and Madrass, was precisely what Dr. James Johnson pointed out six years ago; namely, opium, calomel, venesection, and the warm bath.

"The practice I followed, says Mr. White, was that first recommended by Mr. Johnson, and since by Mr. Corbyn, in which the corner stone and sheet anchor is calomel in a large dose, of fifteen to twenty grains in an adult, according to his strength; and I have had the most manifest proof, both in this disease and formerly *in severe fever and dysentery*, that calomel in this quantity acts as a powerful sedative, often allaying vomiting, and soothing uneasy sensations when no other medicine will produce such beneficial results. I have always combined the calomel with opium."

"The Medical Board at Madras concur with the authorities in Bengal in recommending calomel." *ib.*

The testimonies in favour of venesection are equally strong.

"On admission, says Mr. Burrel, I bled in every instance, in general to a good extent; when universal spasms existed venesection was carried *ad deliquium*, the patients at the same time being in the hot bath at 110.° The spasms were invariably relieved,

* *Ed. Journal, LX.*

nausea and vomiting alleviated, so that the stomach bore the exhibition of calomel in scruple doses, combined with laudanum, which doses were frequently repeated. In short, opium was given under every combination with calomel, and I believe the calomel will be found to rest on most stomachs *per se*. Bleeding has been tried in the native attendants with the same good success."

Mr. Henderson also gives his testimony to the excellent effects of bleeding in this dangerous disease; a measure which, he says, "exceeded his most sanguine expectations."—The same is corroborated by many other practitioners.*

We trust that these authentic documents will induce Dr. Bateman, in this place, as he has been forced to do in many others, to abandon "the doctrine of the schools," in favour of actual facts and the phenomena of Nature.

Rep. 6. In this Report are some useful observations on gastrodynia, or stomach-colic, known in Scotland and Ireland by the name of "water-brash," and the symptoms of which are familiar to all medical men. In one instance of this complaint, under our author, accompanied by great gastric irritability, a numbness and debility of the fingers, resembling the partial paralysis from lead, took place.

"This gradually increased till the whole of both arms became absolutely paralytic, accompanied with great pains in the limbs and back. I must make my acknowledgements to Dr. Hamilton, for the relief which I have been able to afford this patient. The bowels were not greatly constipated; the stools, however, were black, offensive, and of unhealthy consistence; and by frequent exhibitions of about iij gr. of calomel alone (for she bore this when every other medicine was rejected) the stomach was gradually brought to a considerable strength, the pains have ceased, and the elbows and wrists have greatly recovered their motion; the fingers, however, remained considerably paralysed." 57.

In the same Report, Dr. Bateman recommends the external application of *cold water* in arthritis rheumatica; or, as it is termed rheumatic gout.

"This modification of local inflammation, (says Dr. B.) being unconnected with the stomach, seemed to be without any probable danger of *retrogression*, and the event has hitherto accorded with this anticipation."

---

* Mr. Sheppard, in the Second Edition of Dr. Johnson's Work on Tropical Climates, has stated his success in cholera by *venesection* carried to *syncope*, at Rio Janeiro.

We would ask Dr. Bateman, what he means by a *modification* of local inflammation? We presume that it means something *different* from common *local* inflammation; and this difference is, in fact, its connection with the *general* state of the constitution. We have never yet seen a perfectly *local* inflammation in gout or rheumatism, of the acute kind. Nor do we believe that the safest, and consequently the best modes of treatment will ever hinge on local applications alone. We have long been in the habit of using evaporating lotions of a slightly tepid temperature, and gently stimulating nature, as the liq. ammon. acet. or the mist. camphoræ, with a small proportion of spirit, in cases of rheumatic and arthritic inflammations, and with great benefit. But the application of cold water to these inflammations has, to our own knowledge, produced deleterious effects, and we believe it is now seldom used by our most distinguished physicians.

In the 8th Report, [Ed. Journ. Sept. 1806] Dr. Bateman speaks in favourable terms of the nitric acid in jaundice, from gall-stones or inspissated bile.

"The influence of this medicine on the secretion of the liver appears to me, therefore, unquestionable, when taken internally."

Upon the following passage we shall take leave to comment.

"Its agency, when used externally, by immersion, in the manner recommended by Dr. Scott, is in such direct opposition to physiological principle and direct experiment, *which prove the non-absorbing power of the skin*, except by the aid of strong friction, that it is surprising it should have received the countenance of any of the cultivated members of the profession." 67.

Here we find Dr. Bateman on his old *hobby-horse*, the "doctrine of the schools," which has already served him such scurvy tricks, riding full speed against actual facts. Having learnt from Mr. Ellis that the skin does not absorb, he is astonished to find hundreds of accurate and enlightened practitioners believe the evidence of their own senses, respecting the nitro-muriatic acid bath, in opposition to the theory of the above-mentioned gentleman. Dr. Bateman forgets that the experiments of Sir Everard Home, Mr. Brodie, and others, prove that substances can find their way into the blood without the aid of either lacteal or lymphatic absorbents;* and that the theory of non-

---

* *Philosophical Transactions*, 1811-1816.

We would ask Dr. Bateman the following simple question. How absorption by the skin is so far from being settled, that so late as the year before last, Dr. N. L. Young instituted an extensive series of experiments on this subject, all tending completely to refute Mr. Ellis's doctrines.* Yet, upon a doubtful, or rather a positively erroneous hypothesis, Dr. Bateman, without one particle, or even pretence to experience, denies a body of facts observed by hundreds, or rather thousands of medical men, all equally as capable of judging and discriminating as himself! We have had such decisive proofs of the effects of the nitro-muriatic acid externally applied, both in our own persons, and in hundreds of others, that we cannot but admire the *sangfroid* with which Dr. Bateman classes all who can see or believe in these effects, with a Mesmer or a Perkins. But Dr. Bateman will probably find by the event, that this adjudication and the prediction following it, are short-sighted and erroneous.

In the 15th Report [Ed. Journal, July 1808] we have some judicious remarks on dysentery.

"But the irritation which the stimulus of a cathartic medicine excites, in that tender condition of the canal, must be obviated: and this object is best attained by combining an opiate with the cathartic. These cases illustrate that principle in a satisfactory manner; for the patients were all restored to health in a few days, by taking a pill of opium and calomel on two or three alternate nights, and in the day time a solution of magnesia vitriolata with laudanum. The operation of the purgative is not impeded by this combination, but is rendered mild and unirritating; the bowels are emptied without tenesmus, and the natural secretion of the canal is restored. The irritation occasioned by a cathartic medicine is not so completely removed by an opiate given *after* its operation is finished, as is commonly recommended, and the dysenteric symptoms, suspended by this practice, are much more liable to recur." P. 101.

Much false theory and bad practice still obtain in the management of dysentery in this country, among those who have not seen it on an extensive scale, and in aggravated forms in other and less healthy climes. The afflux of blood to the mucous membrane of the intestines, and

---

does Belladonna smeared [not rubbed] on the forehead, produce dilatation of the pupil? One would think that this single *fact* might *open the eyes* of Dr. Bateman to the error of his non-absorbent theory. If he talks about nervous influence in this case, we claim the same argument in the case of nitro-muriatic acid applied to the skin.

* *Med. Chir. Journ.* No. 3, p. 431.

the acrid secretions which are poured into the primæ viæ have led to the erroneous idea that these morbid secretions must be carried off daily by purgatives. But a more enlightened theory, and what is better to the purpose, a more judicious practice, point to a *prevention* of these morbid secretions, which will obviate the pain and the necessity of their removal. Change the balance of the circulation from the abdominal viscera to the surface, by warmth, quietude, and sudorifics;—allay the irritability of the intestinal track by opiates and cretaceous absorbents; and, in this country at least, you will have occasion to do little else, except exhibiting, from time to time, a little castor oil or the neutral salts.

In the 18th Report, [Ed. Journ. April, 1809] Dr. Bateman observes, that hemicrania, or rheumatic face-ache, was invariably aggravated by fomentations — sometimes relieved by Dover's powder; "but the combination of calomel and opium, alone or with the pulvis antimonialis, appeared to be the most successful remedy."

In the same Report, a case of diabetes mellitus is mentioned, which occurred in the person of a poor woman, aged 59, who lived, previously to the attack, three months in a damp cellar, and with little other sustenance than tea. For six months prior to the Report, she had been passing about eleven or twelve pints of melleous water daily, of a sickly violet colour, void of urea, and slightly acidulous. Great benefit was derived from

"A combination of cinchona, uva ursi, and opium, taken three times a day, in the proportion of a scruple of the first, xv grains of the second, and half a grain of the latter in powder." 116.

Under this course, the urine became diminished to the natural quantity, and of the urinous quality; her feverishness disappeared, and her strength and spirits were considerably recruited. On leaving off the medicine, however, the complaint returned. Dr. Ferriar says he cured three confirmed cases of diabetes by the above-mentioned medicine.

In the succeeding Report, [19] there is a striking illustration of the sympathetic influence of the epigastric viscera over the nervous, and through that, over the muscular system. A delicate girl, of eight years of age, was affected with a constant involuntary opening and shutting of the mouth, as if in the act of mastication, accompanied by a languid and spiritless condition of the body, some tension of the epigastrium, and irregularity of the bowels.

"A single purgative, containing three grains of calomel, removed the inordinate action of the maxillary muscles; and in a few days, the healthy complexion and spirits were restored under the use of a decoction of cascarilla."

We shall quote the following passage relative to hemicrania, from the same Report, as it will give us an opportunity of introducing one of the most remarkable cases of the kind on record; a case, the unfortunate victim of which is well known to most of the upper classes of medical society in this metropolis.

"This very painful affection, though not uncommon, has seldom been mentioned by medical writers, or has been confounded with *tic douloureux*.* It consists of a pain near the inner angle of the orbit, beginning in the point of the supra-orbital foramen, and extending a little way on that side of the nose, affecting also the eye, which becomes red, and more sensible to light during the paroxysm. The pain is very acute, and each pulsation of the supra-orbital twig of the artery is painfully felt. The seat of the pain may be generally covered with the point of the finger, and pressure gives momentary relief, although the integuments become tender during the paroxysm, and remain so, in some degree, during the intermission. When the disorder has continued several days, the skin of all that side of the forehead, which is supplied by the supra-orbital nerve, has become tender to the touch. After a duration of two or three hours, sometimes much longer, the pain diminishes, and soon ceases; but it returns again, sometimes most exactly at the same hour on the succeeding day. All the cases that we have seen have been quotidian. The disease differs altogether from the *tic douloureux*, in the regularity of its diurnal periods and intermissions, and in the length of both. In the *tic*, 'the pain (says a writer in the Med. Obs. and Inq.) comes on suddenly, and is excruciating; it lasts but a short time, perhaps a quarter or half a minute, and then goes off; it returns at irregular intervals, sometimes in half an hour, sometimes there are two or three repetitions in a few minutes.' The most decidedly active remedy, according to our observation, is four or five drops of the solution of arsenic, taken twice or three times a day." P. 121.

We do not see any sufficient reason laid down by our author why the foregoing disease should be considered to "differ altogether from the *tic douloureux*." What is tic douloureux but a *neuralgia*, whether the nerve be a branch of the 5th or of any other pair in the body? If the dis-

---

* Sauvages has very well characterised it in the following passage:— "Est morbus cujus præcipuum symptoma dolor est in alterutro capitis latere potissimum ad tempora, frontem, juxta oculos isque vehemens, sæpe periodicus." *Nos. Met.*

ease exhibit some shades of peculiarity determined by the various functions or connections of the nerves affected, this constitutes no essential difference in the pathology, or perhaps the treatment of the complaint. Most of our readers are aware that Dr. Parry attributes this painful disease to an increased vascularity of the neurilema; Mr. Abernethy and his followers, to derangement of the digestive organs; the continental physicians to a morbid *irritation* of the nerve or its covering. A late writer, Mr. Bailey, of Harwich, cannot allow that the disease is always connected with, or preceded by any constitutional disorder; but we shall here throw out a hint that may induce Mr. B. to re-consider the subject. The internal organs are naturally insensible; that is, the intellectual system does not recognize the sensations produced on the organic system in ordinary states of health, or until a *morbid* sensibility is raised in the organs alluded to. Now there may be a considerable *degree* of irritation going on in the organic system, the heart, stomach, and other chylopoietic viscera, before it becomes cognizable to the senses; and this *latent* or nascent irritation may, and every day does, produce various sympathetic pains and derangements of function, at a distance from the primary source of the mischief. We can thus readily perceive how neuralgic affections may be manifested long before the constitutional disease becomes apparent, and that the latter may then be taken for an effect, when it was, in reality, the cause of the former.

A few weeks ago, we were consulted by a gentleman of the law, whose sufferings have induced him to solicit the advice of almost every medical man of the metropolis, whose advice was worth asking. The following sketch of his complaint, therefore, will be readily recognized by numbers of the profession. He is about 45 years of age, tall, thin, and sallow, with a careworn, but intelligent countenance. For more than five years past, he has been harassed with *tic douloureux,* which is confined to a point of indefinite minuteness on the inner ankle of the left foot. Although this point might be covered by a pin's head, no human language can convey an idea of the horrible torture which is there concentrated at each lancination of pain! This unfortunate gentleman can compare it only to what might be supposed to result from a red hot iron dashed through the ankle joint. The stroke is rapid and transient as the lightning's flash, and is in various degrees of severity, from that attempted to be described, down to a slight *twitch*, which would not otherwise be regarded, but as it harrows up the recollection and dread of more severe paroxysms. The number of repetitions of the attack also varies much; from twenty or thirty in a day to two or three. Of late, it is a rare occurrence to pass a whole day without visitations from this enemy of his repose. He informed us, that previous to the accession of this complaint he was remarkably robust, active, healthy, and temperate; that symptoms of indigestion, and flatulent or fetid eructations, preceded the development of the affection, which came on gradually but steadily progressive in its march and aggravation. About two years ago, having gone to Brighton, and there used the warm salt water bath for some time, the TIC, to his inexpressible joy and surprise, totally, or almost totally, disappeared for some months, during which his spirits, strength, and flesh, were beginning to recruit; but alas! this was only a suspension of the disease, for it returned with tenfold violence at an unexpected moment, when he fondly hoped that he was secure from its attacks. It may be proper here to state, that this relapse took place immediately *post coitum,* and that at all times this operation so aggravated the complaint as to make him dread and avoid it, even when the appetite recurred, which was but seldom, in his shattered state of health and mental anxiety.

But the most interesting and instructive phenomenon attending this deplorable case, remains yet to be stated. An *invariable* accompaniment of the torturing stab* at the ancle is a gurgling noise, and uneasy sensation about the sigmoid flexure of the colon, the flatus quickly ascending to the stomach, and there giving rise to an eructation of gas, of no peculiar fœtor, when a temporary security from another plunge is pretty certainly gained. But if the eructation does not immediately take place, the unhappy sufferer is stabbed repeatedly by this invisible dagger till the flatus is dispelled. On this account, the moment he feels the blow at the ancle, whether it be a slight twitch or a most poignant dart, he jumps up on his feet, even if in bed, or at the middle of a meal, or in the most earnest conversation, and pressing his hand with all his force on the sigmoid flexure of the colon, endeavours to extricate the gas with all possible expedition, as he knows, by dire experience, that he will have no security from the ancle affection till the flatus is dislodged.

* We use his own terms in most places; they may not be scientific, but they are sufficiently expressive.

Such is the life of this unfortunate gentleman, whose long sufferings, and minute attention to his complaint, have rendered him not only circumstantially intimate with all its phenomena, but perfectly aware of the various juvantia, or rather the *lædentia;* for although there is scarcely any thing capable of doing him good, there is a long catalogue of things which aggravate his sufferings. These principally consist in dietetics. He would not, for instance, eat a slice of ham, and drink a glass of porter, were he paid one hundred pounds for so doing, such are the terrible effects which would inevitably follow. Almost all the flatulent vegetables aggravate his complaint, and are therefore proscribed from his table. But it would form a long article to detail the natural history of this dreadful affliction; suffice it to say, that he has taken the advice of almost every man of reputation in London, without experiencing one particle of benefit therefrom. The only medicines that ever appeared to mitigate his sufferings were hit on by chance; and these were, *purgatives* and the cinchona. Mercury, sarsa, opium, arsenic, belladonna, and a hundred other remedies, produced no effect. The only *premonitory* sensations he experiences, are those of the dyspeptic character; and he can, on many occasions, feel the twitch dart from the left side of the abdomen to the ancle, just as it is about to inflict the dreaded stab.

He cannot trace the cause to any thing of a moral nature; for his circumstances were easy, and his domestic life happy, till this dire visitation. *Now,* however, the spirits are depressed, and the mental spring is necessarily enfeebled by the corporeal sufferings.

This case is extremely interesting. No finer specimen of tic douloureux was ever presented to view, and nothing can be more clear than that the *fons et origo mali* is seated in the line of the digestive organs. But what is the nature of the evil? Is it a morbid secretion? or, is it some morbid structure? Can there be any extraneous body lodged in some part of the canal, and causing the occasional nervous torture? The channel of communication appears to be the anterior crural nerve. Would a division of this nerve have any effect? No operation has ever been proposed; nor do we believe that any one would be of use. We have yet seen the gentleman but thrice. The first time, he related his history merely, and begged us to *reflect* on it for some days before we prescribed. That we entertain little hopes of a cure need not be specified; yet we think a *conversion* of the disease is not beyond the range of possibility.

Whatever we can learn of this remarkable case shall be communicated on a future occasion.

Meanwhile, our limits are now transgressed; and other toils demand our care. It is an article of our belief, that from every evil some good results—and it ought to be a principle in every man's policy, to *convert* each natural or artificial obstacle to his course, into an engine for the acceleration of its progress. Of this we have just offered a practical illustration. We took up the work reviewed with an expectation of much new and valuable materials for the construction of our Quarterly Number. We were therein disappointed. But as Camper contrived to write an interesting book on the "*shape of a shoe,*" so we trust that we have raised a useful superstructure on the republication of Dr. Bateman's "Reports."

